# Supplementary material for: Spindle architecture constrains karyotype evolution
Source: Nat Cell Biol. 2024 Aug 8;26(9):1496–503. doi: 10.1038/s41556-024-01485-w (PMC11392806; doi:10.1038/s41556-024-01485-w)
Supplement: Supplementary file 1 — Reporting Summary [file 41556_2024_1485_MOESM1_ESM.pdf]

nature portfolio

Corresponding author(s): Gautam Dey
Gavin Sherlock
Jana Helsen

Last updated by author(s): Jul 15, 2024

Reporting Summary

Nature Portfolio wishes to improve the reproducibility of the work that we publish. This form provides structure for consistency and transparency in reporting. For further information on Nature Portfolio policies, see our Editorial Policies and the Editorial Policy Checklist.

Statistics

For all statistical analyses, confirm that the following items are present in the figure legend, table legend, main text, or Methods section.

n/a	Confirmed	
☐	☒	The exact sample size (*n*) for each experimental group/condition, given as a discrete number and unit of measurement
☐	☒	A statement on whether measurements were taken from distinct samples or whether the same sample was measured repeatedly
☐	☒	The statistical test(s) used AND whether they are one- or two-sided<br>*Only common tests should be described solely by name; describe more complex techniques in the Methods section.*
☐	☒	A description of all covariates tested
☐	☒	A description of any assumptions or corrections, such as tests of normality and adjustment for multiple comparisons
☐	☒	A full description of the statistical parameters including central tendency (e.g. means) or other basic estimates (e.g. regression coefficient) AND variation (e.g. standard deviation) or associated estimates of uncertainty (e.g. confidence intervals)
☐	☒	For null hypothesis testing, the test statistic (e.g. *F*, *t*, *r*) with confidence intervals, effect sizes, degrees of freedom and *P* value noted<br>*Give P values as exact values whenever suitable.*
☒	☐	For Bayesian analysis, information on the choice of priors and Markov chain Monte Carlo settings
☒	☐	For hierarchical and complex designs, identification of the appropriate level for tests and full reporting of outcomes
☒	☐	Estimates of effect sizes (e.g. Cohen's *d*, Pearson's *r*), indicating how they were calculated

Our web collection on statistics for biologists contains articles on many of the points above.

Software and code

Policy information about availability of computer code

Data collection	Gen5 software (version 3.11, BioTek) was used to control the BioTek Epoch2 microplate readers (Agilent) during growth rate measurements. For live-cell microscopy the microscope was controlled by cellSens Dimension software (version 3.2, Olympus).
Data analysis	Maximum growth rates were determined using the gcplyr package in R (version 1.5.2). Fiji (ImageJ2 version 2.9.0) was used for basic image processing (cropping, z-stack projections, scaling, LUT selection), and for measuring spindle curvature and SPB distance over time. For analysis of sequencing data, the quality of the reads was assessed using FastQC version 0.11.9 (Babraham Bioinformatics), and Nextera transposase sequences were trimmed using Trim galore! version 0.6.7 (Babraham Bioinformatics). Trimmed reads were mapped to the reference S288c genome (version R64) using bwa-mem version 0.7.17 with default settings. Indels and SNVs were called using GATK version 4.2.6.1, using HaplotypeCaller and default settings. High-quality SNVs and indels were verified by manual curation in IGV version 2.12.3.

For manuscripts utilizing custom algorithms or software that are central to the research but not yet described in published literature, software must be made available to editors and reviewers. We strongly encourage code deposition in a community repository (e.g. GitHub). See the Nature Portfolio guidelines for submitting code & software for further information.

Data

Policy information about availability of data

All manuscripts must include a data availability statement. This statement should provide the following information, where applicable:

- Accession codes, unique identifiers, or web links for publicly available datasets
- A description of any restrictions on data availability
- For clinical datasets or third party data, please ensure that the statement adheres to our policy

All images used for data analysis are available at doi.org/10.6084/m9.figshare.c.6890251.v2. Sequencing data were deposited on ENA under accession number PRJEB67700. Raw measurements used for plotting can be found in Source Data. Source data have been provided in Source Data. All other data supporting the findings of this study are available from the corresponding author on reasonable request.
Sequencing reads were mapped to the reference S288c genome (version R64, NCBI: GCF_000146045.2).

Research involving human participants, their data, or biological material

Policy information about studies with human participants or human data. See also policy information about sex, gender (identity/presentation), and sexual orientation and race, ethnicity and racism.

Reporting on sex and gender	Not applicable.
Reporting on race, ethnicity, or other socially relevant groupings	Not applicable.
Population characteristics	Not applicable.
Recruitment	Not applicable.
Ethics oversight	Not applicable.

Note that full information on the approval of the study protocol must also be provided in the manuscript.

Field-specific reporting

Please select the one below that is the best fit for your research. If you are not sure, read the appropriate sections before making your selection.

☒ Life sciences ☐ Behavioural & social sciences ☐ Ecological, evolutionary & environmental sciences

For a reference copy of the document with all sections, see nature.com/documents/nr-reporting-summary-flat.pdf

Life sciences study design

All studies must disclose on these points even when the disclosure is negative.

Sample size	No sample-size calculations were performed. Sample sizes were chosen to be comparable to published manuscripts within the field. Growth measurements and experimental evolution (doi: 10.1093/molbev/msaa172), live-cell microscopy (doi:10.1016/j.devcel.2019.01.018).
Data exclusions	For population growth rate measurements and single-cell microscopy experiments, experiments were only deemed successful if the control strains that were included in each experiment behaved as they should. 2 out of 14 population growth rate measurements were excluded (and later repeated successfully) because of this reason, and 0 microscopy experiments. For population growth rate measurements, single measurements for which the gcplyr package failed to calculate a growth rate (usually because of unexpected spikes in the growth curves due to e.g. bubble formation during incubation) were removed. For single cell microscopy, cells for which the SPB signal could not be reliably identified for 3 or more sequential frames were removed. From the resulting data, nothing was excluded from the analyses (e.g. no outliers were removed). All raw measurements can be found in the Source Data.
Replication	Population growth experiments were each set up with 16 independent replicates, 20 independent cells were chosen for each SPB tracking microscopy experiment, 50 cells were chosen for spindle curvature measurements, 56 independent replicate populations were established for each genotype in the evolution experiments, of which 1 clone from 4 (16chr), 4 (8chr), and 8 (3chr) populations were sequenced and 16 (16chr), 16 (8chr), and 56 (3chr) were checked for ploidy. Western blots were performed in duplicate using independent experiments (both successful).
Randomization	For population-level experiments, such as growth measurements, cell growth before Western blots, and experimental evolution, randomization is not relevant as yeast cultures are uniform on a population level. For single-cell microscopy experiments, random dividing cells were chosen for further analysis from overview images of asynchronous populations.
Blinding	Where possible, data were analyzed using semi-automated methods. In cases where manual filtering or correction was incorporated, same criteria were applied to all strains or treatments under investigation. During the experiments, blinding was not possible as each experiment was performed by an individual investigator who was aware of the experimental groups and treatments.

Reporting for specific materials, systems and methods

We require information from authors about some types of materials, experimental systems and methods used in many studies. Here, indicate whether each material, system or method listed is relevant to your study. If you are not sure if a list item applies to your research, read the appropriate section before selecting a response.

Materials & experimental systems

n/a	Involved in the study
☐	☒ Antibodies
☒	☐ Eukaryotic cell lines
☒	☐ Palaeontology and archaeology
☒	☐ Animals and other organisms
☒	☐ Clinical data
☒	☐ Dual use research of concern
☒	☐ Plants

Methods

n/a	Involved in the study
☒	☐ ChIP-seq
☒	☐ Flow cytometry
☒	☐ MRI-based neuroimaging

Antibodies

Antibodies used

Expansion microscopy: YL1/2 anti-α-tubulin (rat) (1:25), gift from Gislene Pereira [COS Heidelberg, Germany]); goat anti-mouse-IgG coupled to Alexa Fluor 488 (Invitrogen A11029)(1:500). Western blot: mouse anti-HA antibody (Cat. No. 26183, Invitrogen)(1:1000), anti-actin antibody (Cat. No. MAB1501R, Chemicon)(1:1000), HRP-conjugated goat anti-mouse secondary antibody (Cat. No. 31430, Invitrogen)(1:10000).

Validation

Expansion microscopy: this antibody combination was successfully used for yeast in doi: 10.1242/jcs.260240.
Western blot: anti-HA: the antibody has been used successfully in Western blot and immunoprecipitation applications.(e.g. in budding yeast: doi: 10.26508/lsa.202201642); anti-actin: validated for use in ELISA, IC, IH, IH(P), WB for the detection of actin (e.g. in budding yeast: doi: 10.3390/ijms21249574); HRP goat anti-mouse: Product # 31430 has been successfully used in Western blot, IHC and IP applications (e.g. in budding yeast: doi: 10.3390/ijms21249574).

Plants

Seed stocks

NA

Novel plant genotypes

NA

Authentication

NA
