# Supplementary figures and images for: Spindle architecture constrains karyotype evolution
Source: Nat Cell Biol. 2024 Aug 8;26(9):1496–503. doi: 10.1038/s41556-024-01485-w (PMC11392806; doi:10.1038/s41556-024-01485-w)

**Fig 2d**

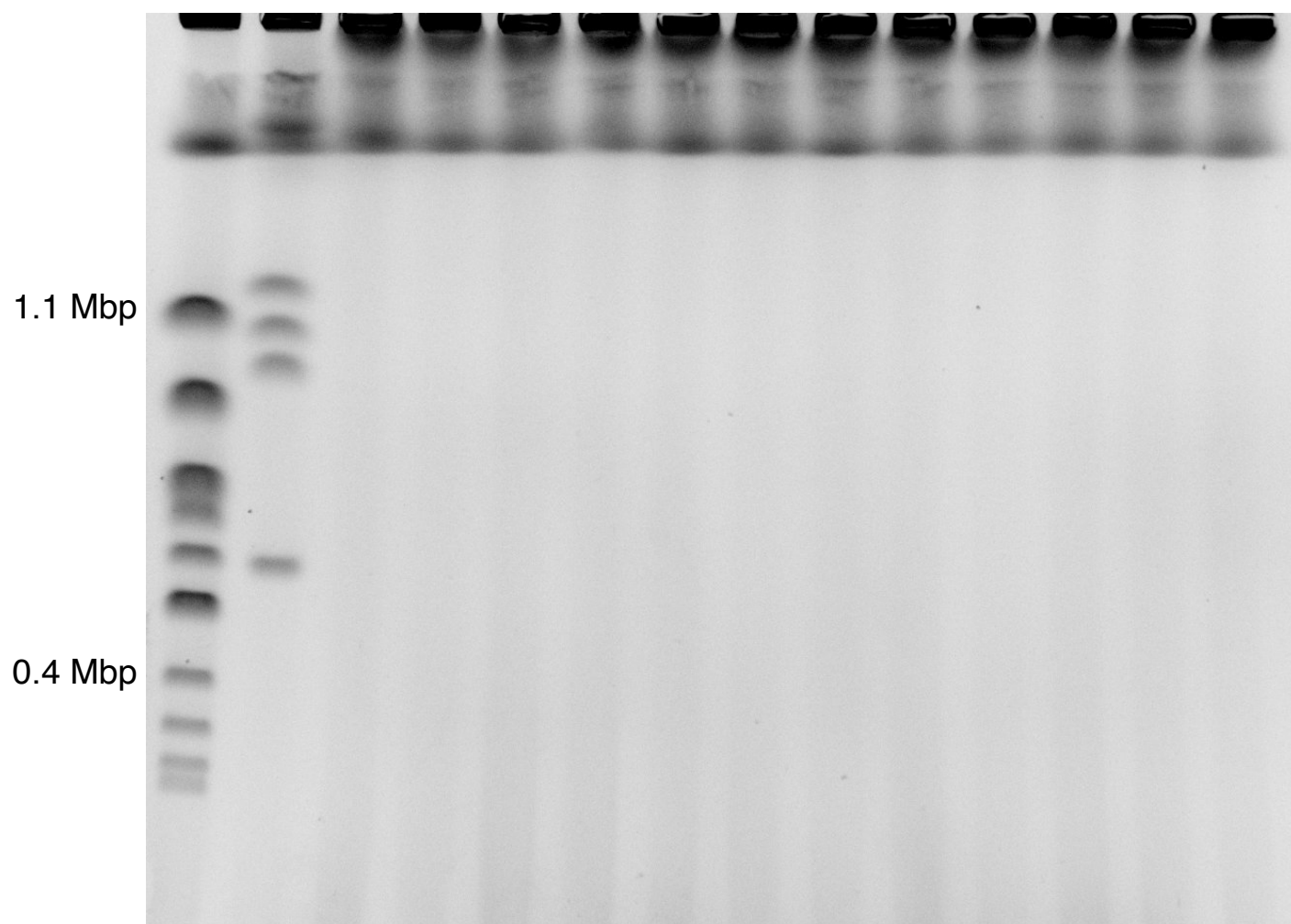

Supplement: Supplementary file 6 — Unprocessed gels. [file 41556_2024_1485_MOESM6_ESM.pdf]

**Fig 5d - wt**

**Pds1**

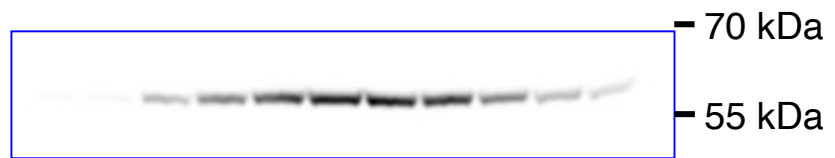

**Actin**

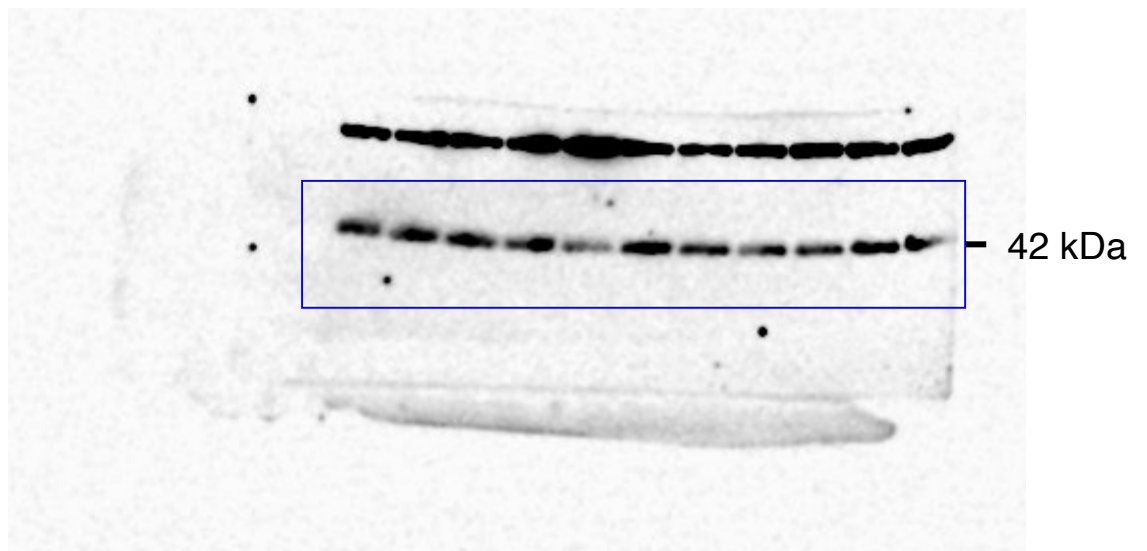

**Ponceau S**

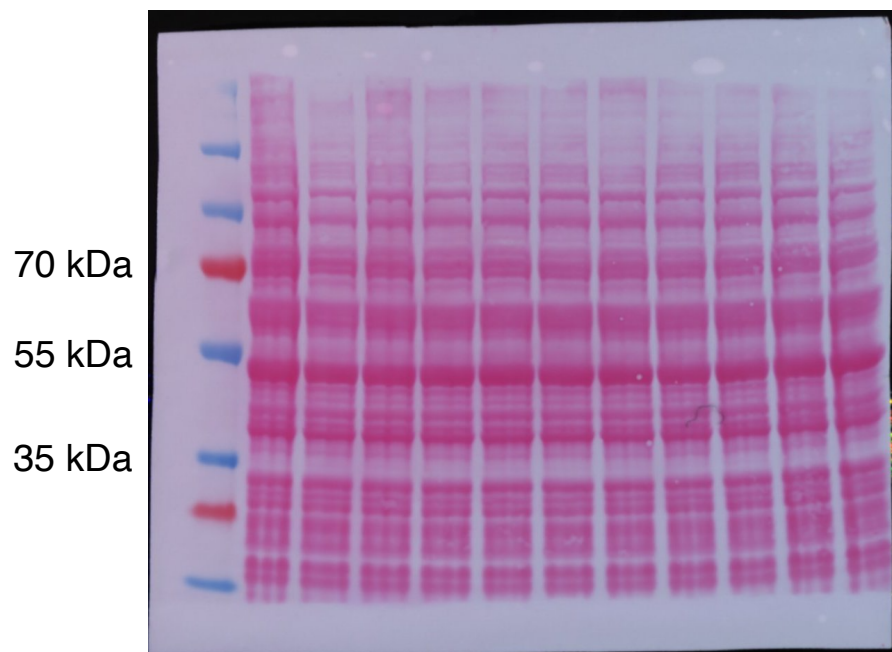

**Fig 5d - 3chr**

**Pds1**

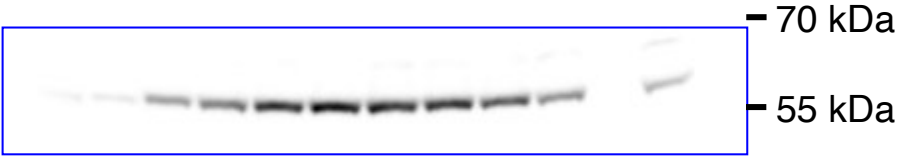

**Actin**

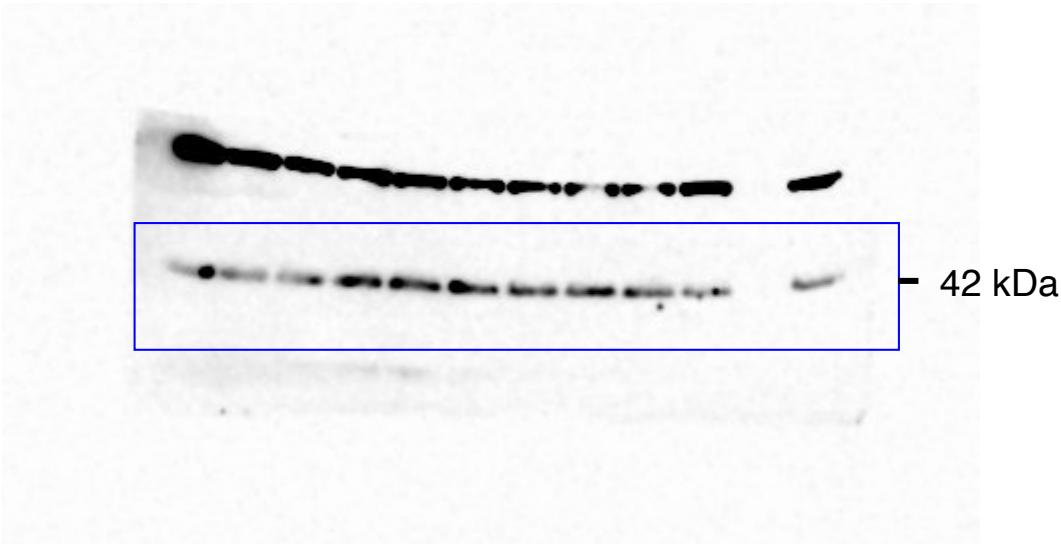

**Ponceau S**

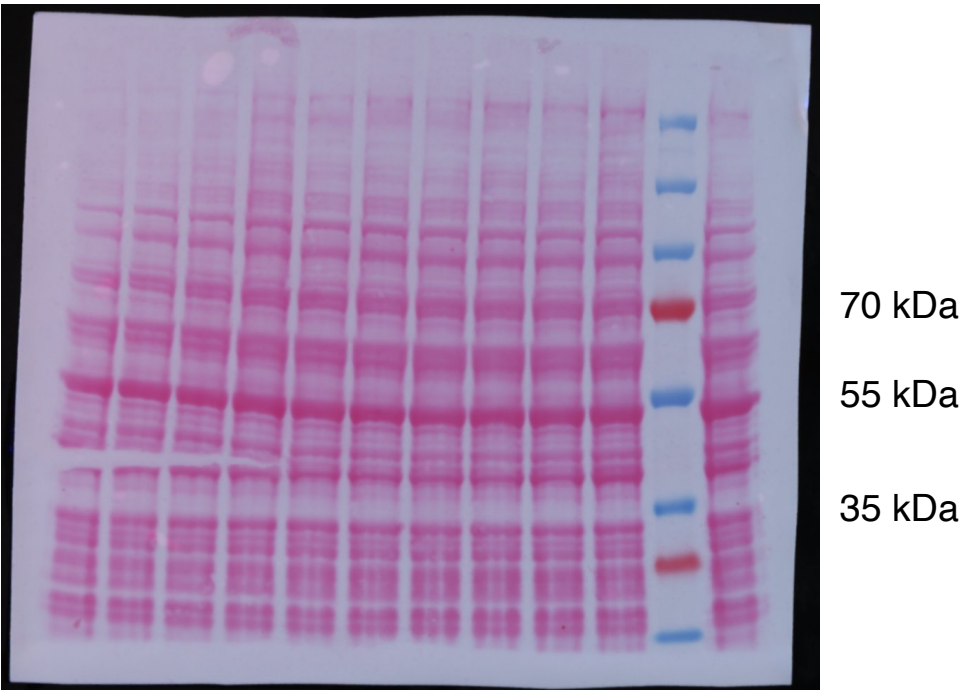

Supplement: Supplementary file 10 — Unprocessed western blots. [file 41556_2024_1485_MOESM10_ESM.pdf]

**Fig S2 - gel 2**

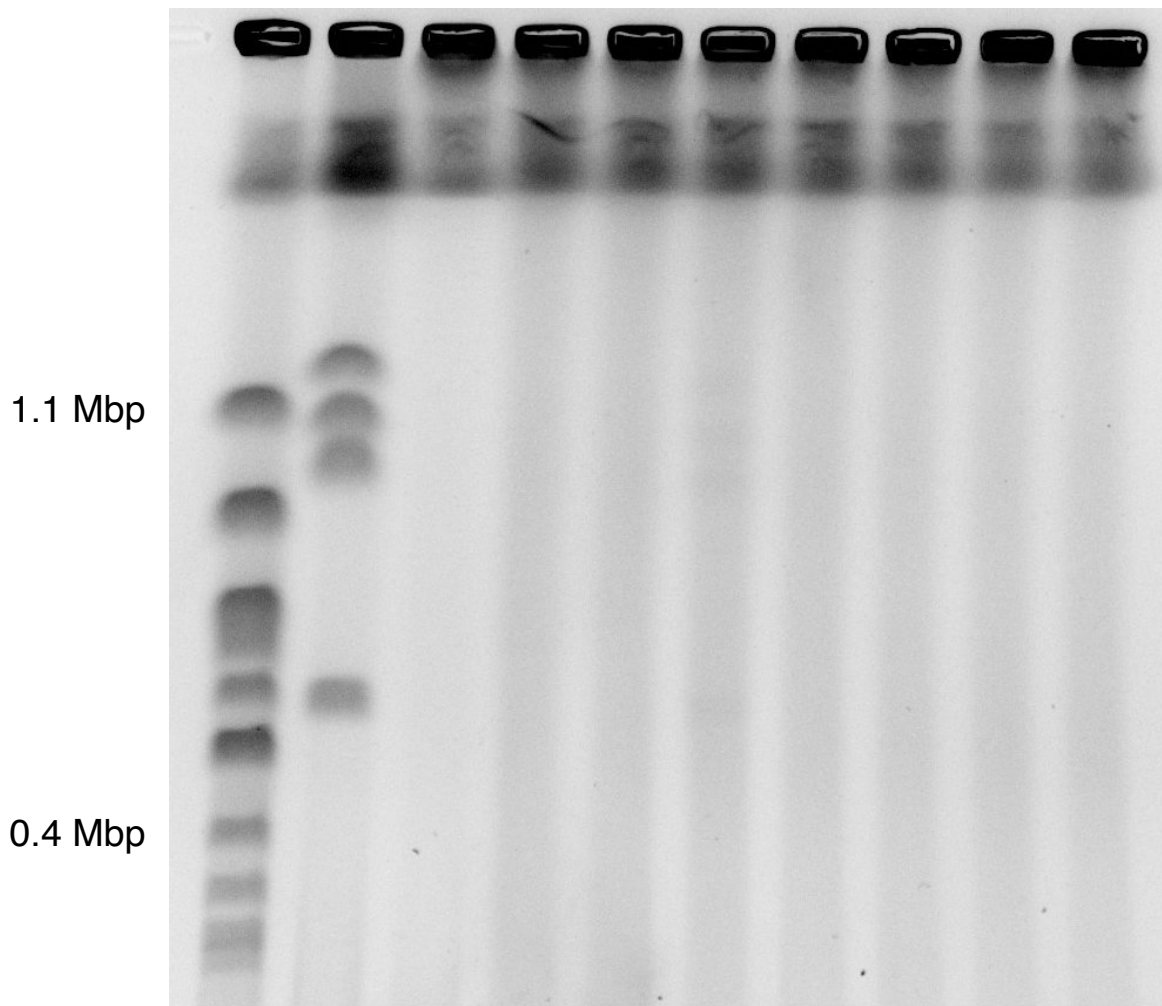

**Fig S2 - gel 3**

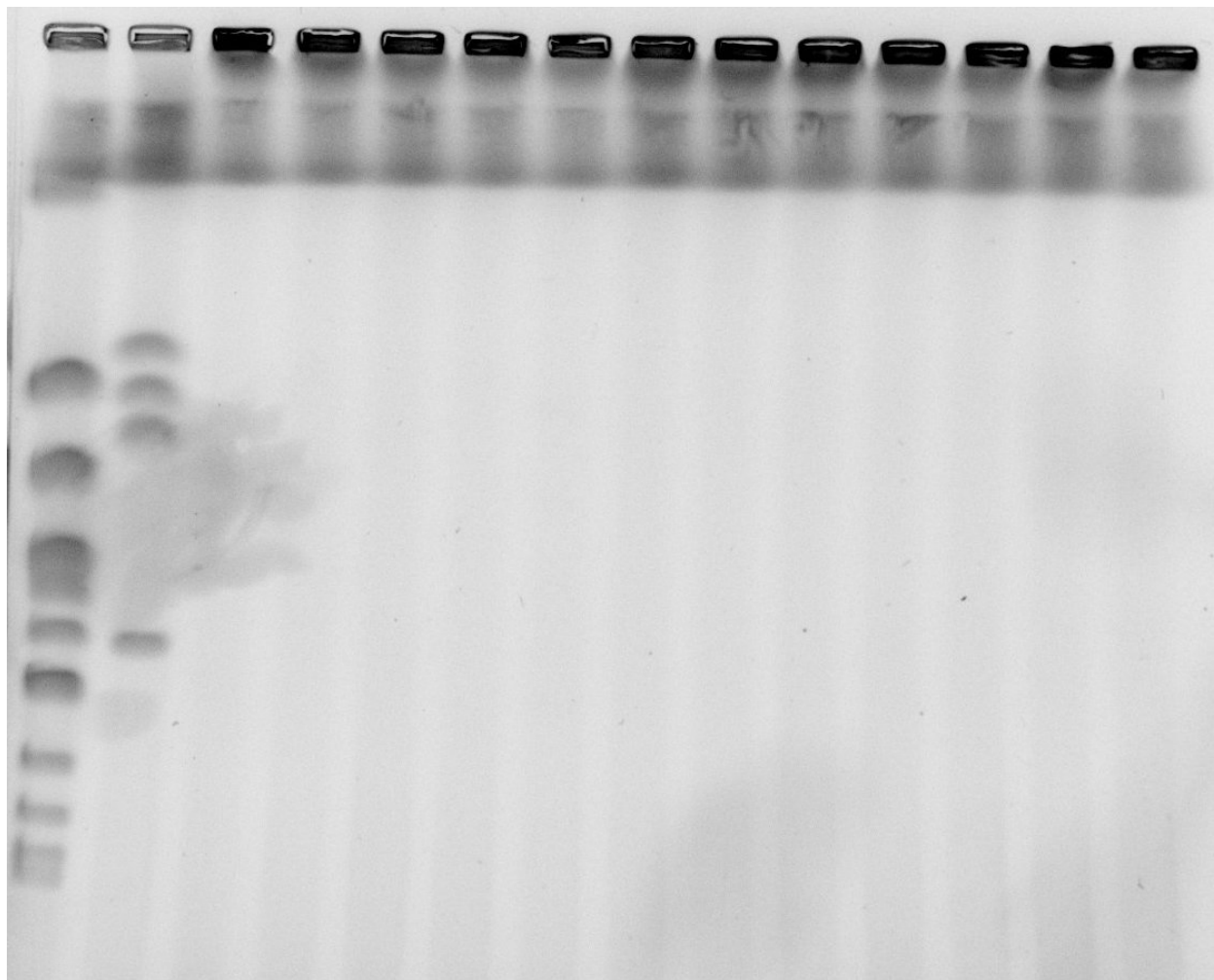

**Fig S2 - gel 4**

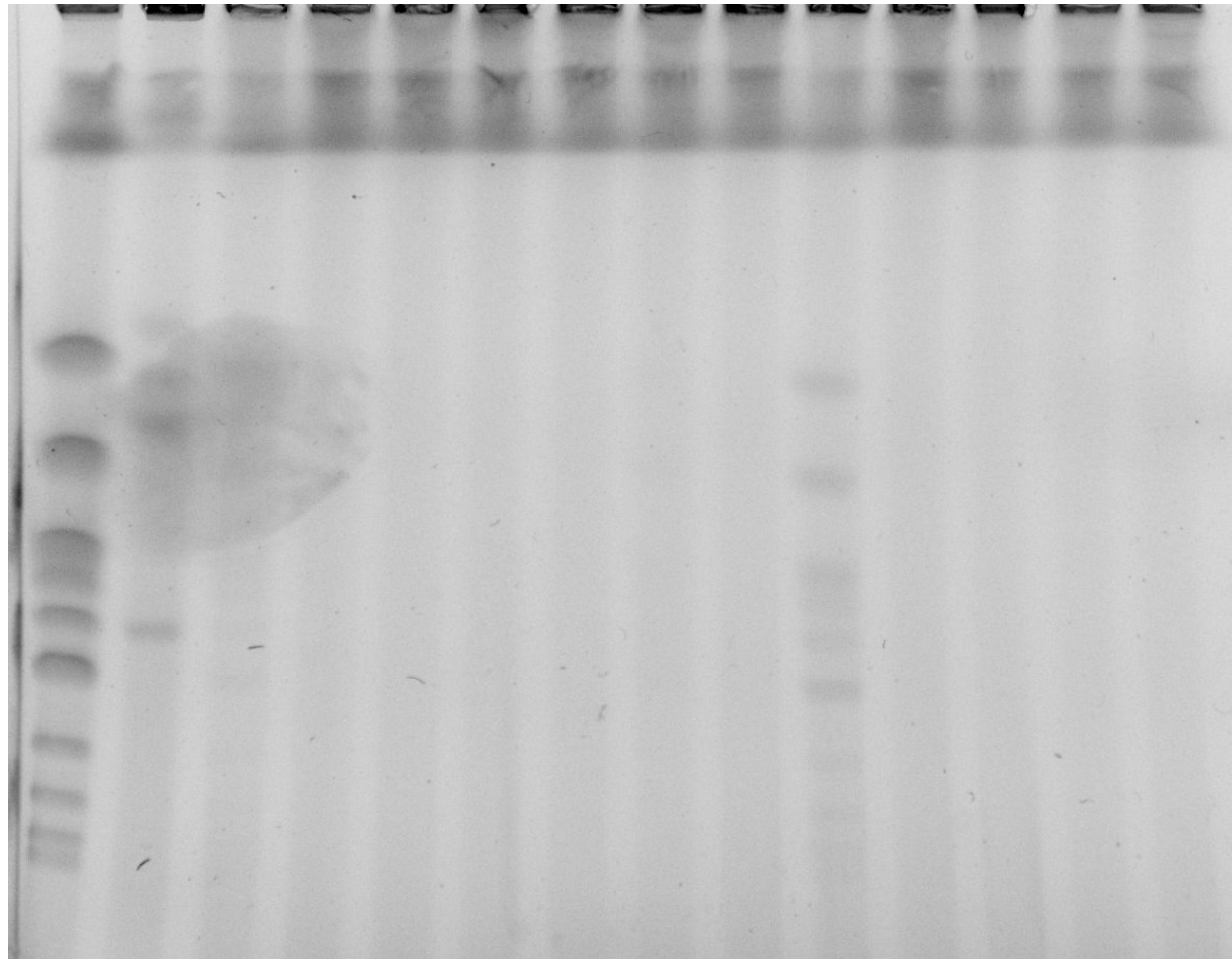

**Fig S2 - gel 5**

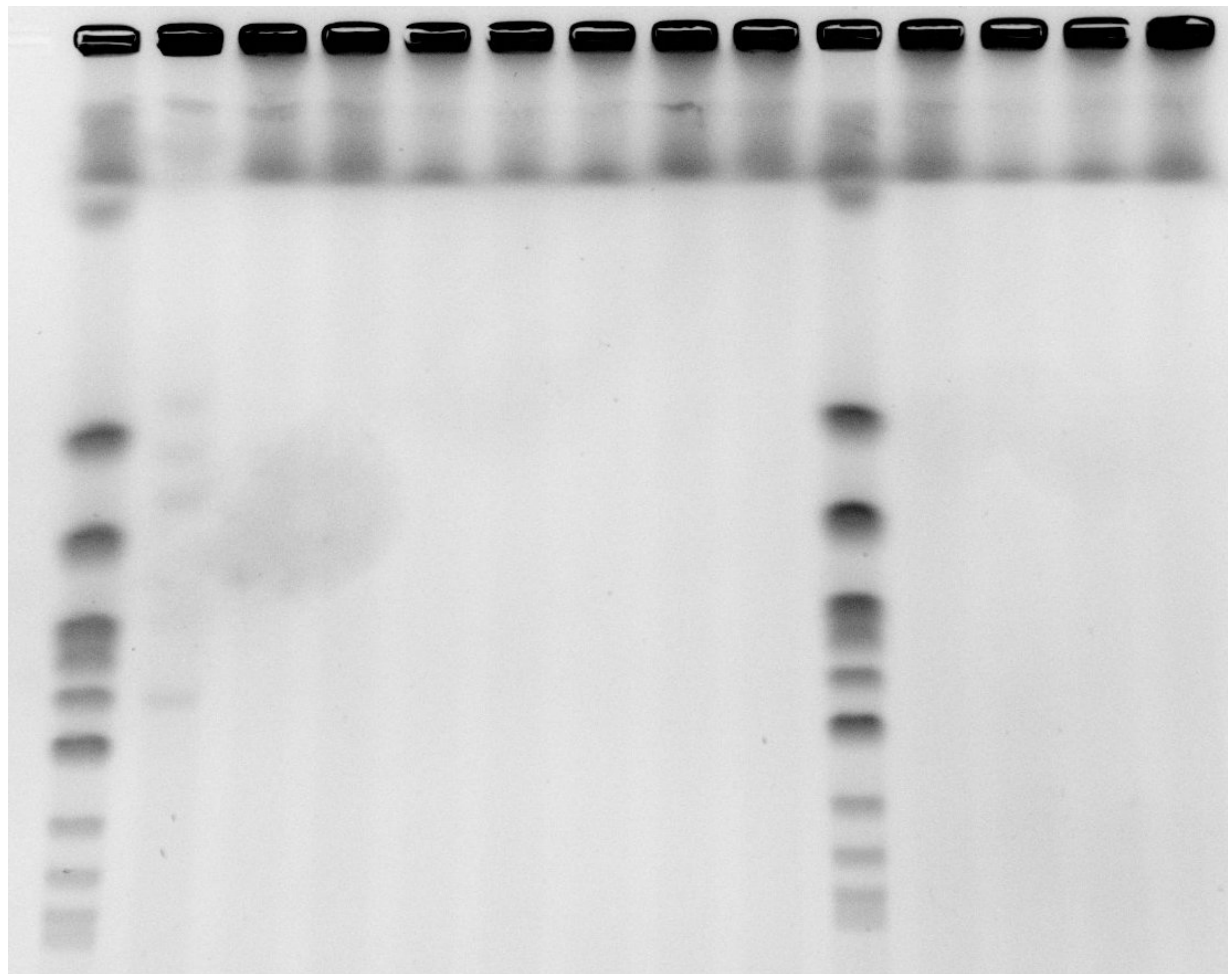

Supplement: Supplementary file 12 — Unprocessed gels. [file 41556_2024_1485_MOESM12_ESM.pdf]

**Fig S5f - wt**

**Pds1**

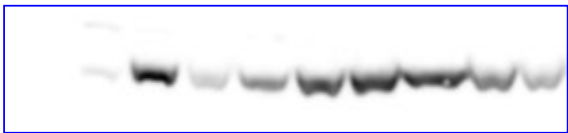

– 70 kDa

– 55 kDa

**Ponceau S**

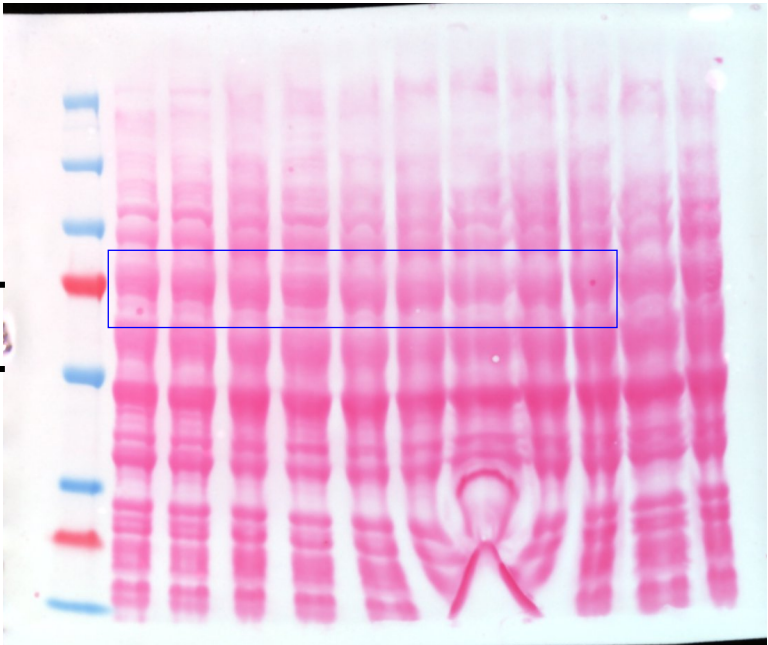

70 kDa –

55 kDa –

**Fig S5f - 3 chr**

**Pds1**

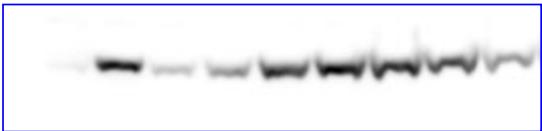

– 70 kDa

– 55 kDa

**Ponceau S**

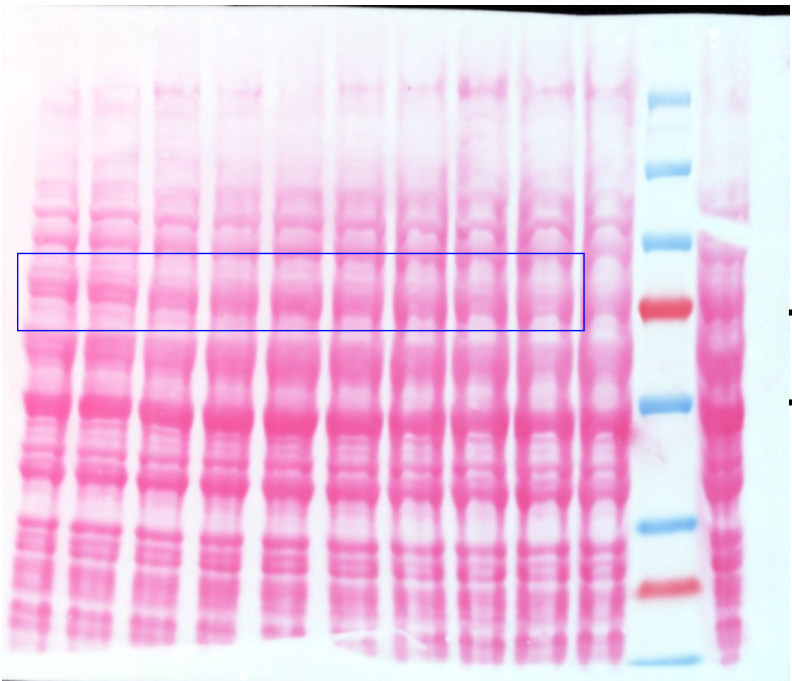

– 70 kDa

– 55 kDa

Supplement: Supplementary file 16 — Unprocessed western blots. [file 41556_2024_1485_MOESM16_ESM.pdf]
